# Gain-of-function nature of Cav1.4 L-type calcium channels alters firing properties of mouse retinal ganglion cells

**DOI:** 10.1080/19336950.2015.1078040

**Published:** 2015-08-14

**Authors:** Dagmar Knoflach, Klaus Schicker, Martin Glösmann, Alexandra Koschak

**Affiliations:** 1 Medical University Vienna, Center for Physiology and Pharmacology; Department of Neurophysiology and -pharmacology; Vienna, Austria; 2 University of Veterinary Medicine, Vetcore; Vienna, Austria; 3 University of Innsbruck, Institute of Pharmacy, Pharmacology and Toxicology; Innsbruck, Austria

**Keywords:** channelopathy, congenital stationary night blindness, contrast sensitivity, L-type calcium channel, multielectrode array recording, type 2

## Abstract

Proper function of Cav1.4 L-type calcium channels is crucial for neurotransmitter release in the retina. Our understanding about how different levels of Cav1.4 channel activity affect retinal function is still limited. In the gain-of-function mouse model Cav1.4-IT we expected a reduction in the photoreceptor dynamic range but still transmission toward retinal ganglion cells. A fraction of Cav1.4-IT ganglion cells responded to light stimulation in multielectrode array recordings from whole-mounted retinas, but showed a significantly delayed response onset. Another significant number of cells showed higher activity in darkness. In addition to structural remodeling observed at the first retinal synapse of Cav1.4-IT mice the functional data suggested a loss of contrast enhancement, a fundamental feature of our visual system. In fact, Cav1.4-IT mouse retinas showed a decline in spatial response and changes in their contrast sensitivity profile. Photoreceptor degeneration was obvious from the nodular structure of cone axons and enlarged pedicles which partly moved toward the outer nuclear layer. Loss of photoreceptors was also expressed as reduced expression of proteins involved in chemical and electrical transmission, as such metabotropic glutamate receptor mGluR6 and the gap junction protein Connexin 36. Such gross changes in retinal structure and function could also explain the diminished visual performance of CSNB2 patients. The expression pattern of the plasma-membrane calcium ATPase 1 which participates in the maintenance of the intracellular calcium homeostasis in photoreceptors was changed in Cav1.4-IT mice. This might be part of a protection mechanism against increased calcium influx, as this is suggested for Cav1.4-IT channels.

## Introduction

Human genetic analyses have been indicating an essential role for Cav1.4 L-type calcium channels (LTCCs) in vision for at least 2 decades.^1,2^ Since then a multitude of functionally different mutations in the human *CACNA1F* gene encoding Cav1.4 LTCCs have been associated with visual disorders, including congenital stationary night blindness type 2 (CSNB2) (for a review see refs^3,4^). Cav1.4 channels, which are mainly expressed at retinal photoreceptor synapses and probably also in bipolar cells^5,6^ allow sustained calcium influx at photoreceptor synaptic terminals due to their unique specialized inactivation properties.^7^ Therefore proper function of Cav1.4 channels is crucial for neurotransmitter release at the first retinal synapse,^8^ as evidenced also by electroretinogram (ERG) abnormalities that are observed in human CSNB2 families.^9^ Based on ERG recordings adult mice carrying the Cav1.4 gain-of-function mutation I745T (Cav1.4-IT^6^) have recently been reported to serve as a specific model for the functional phenotype seen in CSNB2 patients carrying the respective mutation.^10^ Liu and Regus-Leidig and their colleagues reported differences in photoreceptor synapse formation of Cav1.4 deficient (Cav1.4-KO^6,11^) and Cav1.4-IT mice in a direct comparison.^12,13^ Both mouse models showed defects in ribbon synapse formation. Morphological alterations including ribbon shape were more severe in Cav1.4-KO^11,12,13,14^ compared to Cav1.4-IT mice which still showed some horseshoe-shaped ribbons.^10,12,13^ Ultrastructural data^13^ inferred that remaining photoreceptor synapses are largely intact, while many terminals of cone photoreceptors contained free-floating ribbons; a phenotype similar to Bassoon mutant animals.^15^ Lack of visual activity at the level of ganglion cells^8^ (also this study) and in the superior colliculus of the brain^8^ was shown for Cav1.4-KO animals. However, mutant nob-2 mice, which express residual Cav1.4 due to alternative splicing,^16^ showed visual ganglion cell responses but with a reduced dynamic range in ON-center cells.^17^ Deeper insight into the function of Cav1.4 is still lacking. The marked leftward shift in the activation curve found in heterologous expressed Cav1.4-IT channels^18^ would cause a significant increase in calcium influx during illumination. At the same time increase upon depolarization during darkness would be diminished, leading to a reduced dynamic range in the photoreceptor. Ganglion cells as the output cells of the retina might preserve these changes in dynamic range and convey it to higher brain areas.

## Results

### Defects in light-induced activity of Cav1.4-IT mouse retinas

Functional studies previously performed in a heterologous expression system showed a strong hyperpolarizing shift in the voltage-dependence of activation in Cav1.4 channels carrying the gain-of-function missense mutation I745T (Cav1.4-IT,^18^). We therefore hypothesized that such gain in Cav1.4 activity results in a markedly increased calcium influx already during light stimulation. This would decrease the dynamic range of fractional channel opening in a photoreceptor between dark and light conditions, inducing a change in glutamate release, and further affect signaling to ganglion cells as the final output neuron of the retina to the brain. To test this hypothesis, we performed multielectrode array (MEA) analyses of isolated retinas from wild type and mutant mice activated with different visual stimuli.

**Figure 1.**
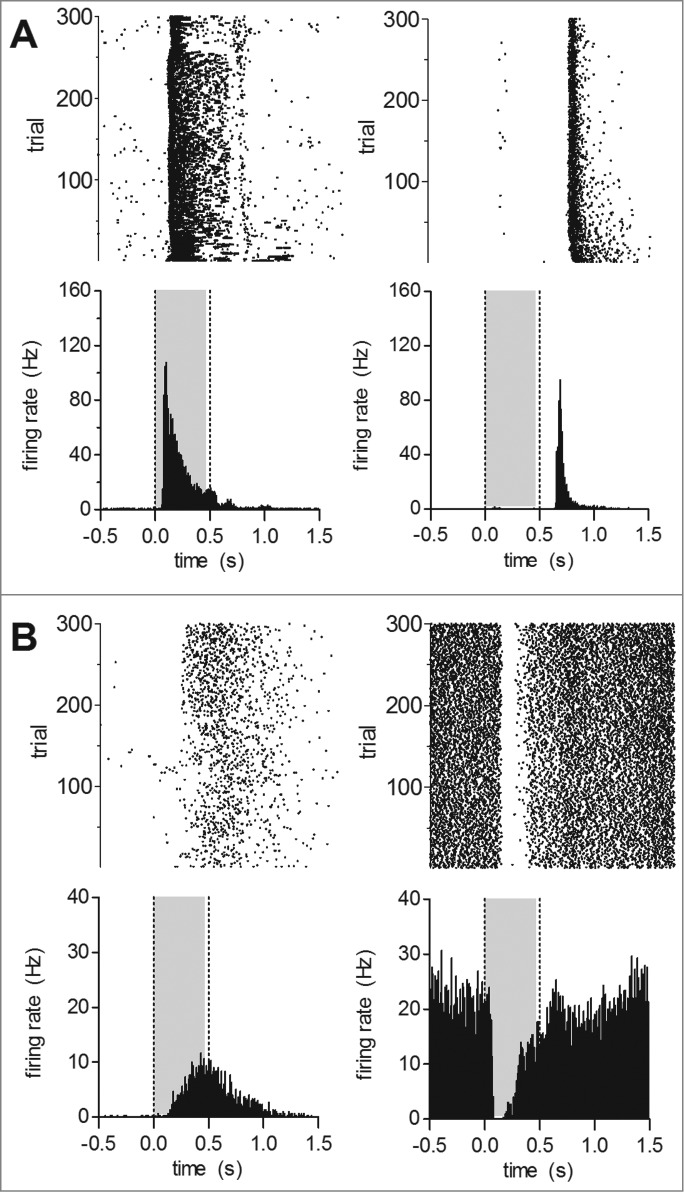
Light induced activity in response to full-field stimuli in wild type and Cav1.4-IT retinas. Short 0.5 s light pulses (gray shading) followed by 1.5 s dark periods were presented 300 times. Upper row: raster plots, lower row: peri-stimulus time histograms (bin size = 10 ms). Representative examples of response patterns found in (**A**) wild type: ON cells (left, cell number K488ffe48–01), OFF cells (right, cell number K551ffe51–03) (**B**) Cav1.4-IT: ON cells (left, cell number K564ff84–03), ‘suppressed’ cells (right, cell number K564ff37–01).

Using full-field flashes we recorded wild type and Cav1.4-IT responses to light increments and decrements. Exemplar responses to full-field stimuli (0.5 s long, presented every 2 s) are shown in **Figure 1**. In three wild type animals we identified 212 single units out of which 210 cells (99%) reacted to light stimuli. Typical ON responses in the wild type were indicated by the increase in mean firing rate upon the transition from darkness to light (**Fig. 1A**, left); OFF responses were characterized by a transient increase in firing rate during transitions from light to darkness (**Fig. 1A**, right). The majority of recorded units demonstrated an ON response (45.6 ± 1.5%, per animal), whereas OFF responses were seen in only 13.7 ± 2.0% of the cells. 38.6 ± 0.8% showed an apparent ON-OFF response type. In contrast only about 60% of units from 4 Cav1.4-IT retinas were light reactive. Three units (1.4 ± 1.4%) showed an OFF response whereas the number of cells responding to light onset seemed preserved (43.8 ± 5.2%). Remarkably, one-fifth of the cells (19.2 ± 1.9% per animal) demonstrated a significantly higher discharge rate in darkness (Cav1.4-IT: 21.0 ± 4.8 Hz, n = 35; F = 4.633; p < 0.001 vs. wild type ON (n = 93), t = 5.097; p < 0.05 vs. wild type OFF (n = 26), t = 3.030; one way ANOVA with Bonferoni post hoc test). This activity was reduced ('suppressed') upon light exposure (**Fig. 1B**, right). Such behavior was only rarely seen in wild type retinas where only 3 units (1.3 ± 1.3%) displayed such a response, similar to previous reports.^19^

**Figure 2.**
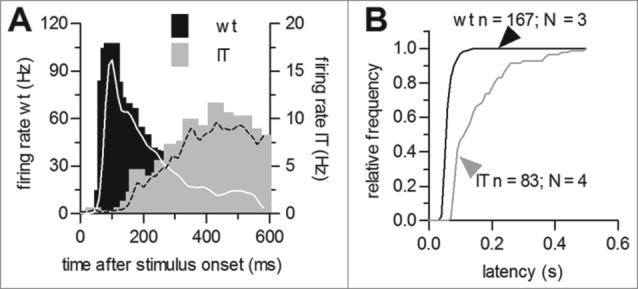
Stimulus response latency of ON-responses in wild type and Cav1.4-IT retinas. (**A**) Comparison of representative examples for peri-stimulus time histograms of wild type (black) and Cav1.4-IT (gray) mouse ganglion cells. Lines represent a smoothed version using a 30-ms Gaussian window function. (**B**) Distributions of wild type (black) and Cav1.4-IT mouse (gray) ganglion cell stimulus response latencies.

We observed a clear difference in the stimulus-response latency of the ON component in Cav1.4-IT retinas (**Fig. 1B**, left). The latency of the response was almost tripled in mutant compared to wild type retinas (in [ms]: wild type: 63.8 ± 1.3, n = 167, Cav1.4-IT: 153.6 ± 10.3, n = 83, p < 0.001, t = 8.649; Students t-test; **Fig. 2**). Thus, the mutation affected the dynamic activity of ganglion cells suggesting that this would cause a reduced sensitivity to light. For comparison we also exposed retinas of Cav1.4-KO mice to the full-field illumination regime. However, all 135 cells that we recorded from 4 Cav1.4-KO retinas lacked response to any light stimulus (data not shown).

**Figure 3.**
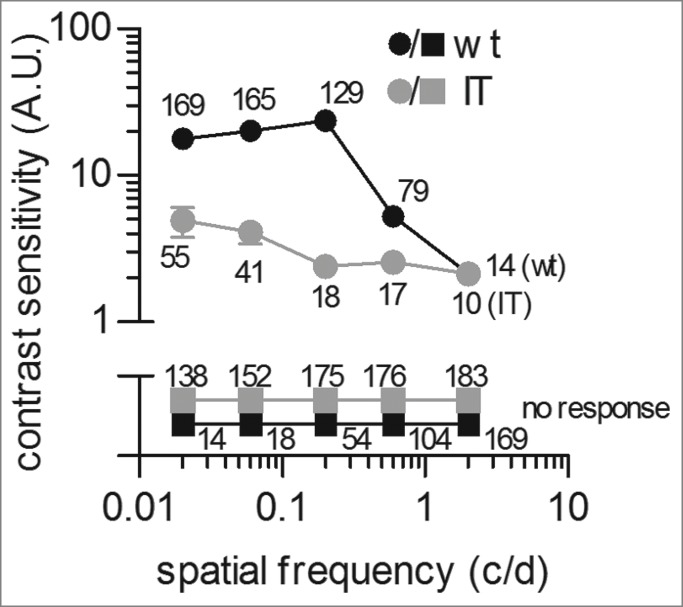
Measurement of contrast sensitivity in isolated wild type and Cav1.4-IT mouse retinas. Retinas were adapted for 20 min to a uniform mid gray stimulus (6.2 mWm^−2^) before presenting a series of 10 s drifting sinusoidal gratings. Spatial frequencies of 0.02, 0.06, 0.2, 0.6 and 2 c/d were presented at contrast values of 0.022, 0.047, 0.1, 0.22 and 0.47 with a constant drift frequency of 1.5 Hz. Spike trains from 10 repeats were averaged for each combination of spatial frequency and contrast. Contrast sensitivity curves show the reciprocal of the lowest contrast needed for detection at each spatial frequency. Data were collected from 3 wild type (black) and 3 Cav1.4-IT (gray) mice. Numbers indicate the total of units that showed a response (circles) or no response (squares) at the indicated spatial frequency.

Next, we investigated the contrast sensitivity (CS) function of isolated wild type and Cav1.4-IT retinas using sinusoidal gratings (**Fig. 3**). The CS function illustrates which contrast can be seen at a range of spatial frequencies (i.e. the number of sine gratings in 1° of visual angle). Therefore gratings were presented at 25 different combinations of spatial frequency and contrast^20^ at a constant drifting frequency of 1.5 Hz (reported to be in the optimal range *in vivo*^21^). The CS function of wild type animals showed the highest sensitivity at 0.2 c/d while at 2 c/d we only rarely detected modulation of firing rate in response to the drifting grating. This finding compared well to data from animal studies in which about 0.1 c/d were reported as the optimal spatial frequency^21^ even in the absence of the dioptric (light-refractive) apparatus of the mouse eye. In contrast, the CS curve of Cav1.4-IT mice was reduced over a wide range of spatial frequencies tested (**Fig. 3**). Thus, Cav1.4-IT retinas showed no sensitivity to finely spaced gratings and even only diminished response on coarsely spaced gratings at maximal contrast. The maximal Cav1.4-IT response was elicited at a spatial frequency of 0.02 c/d while the sensitivity was reduced about 10-fold at 0.2 c/d. These results showed that the Cav1.4 gain-of-function mutation changed the spatiotemporal properties of Cav1.4-IT mouse retinas causing a reduction of contrast sensitivity. In fact, reduced visual acuity – as tested in eye exams using high contrast letters in Snellen charts - is a major complaint in CSNB2 patients.^22^

### Degeneration in Cav1.4-IT mouse retinas

**Figure 4.**
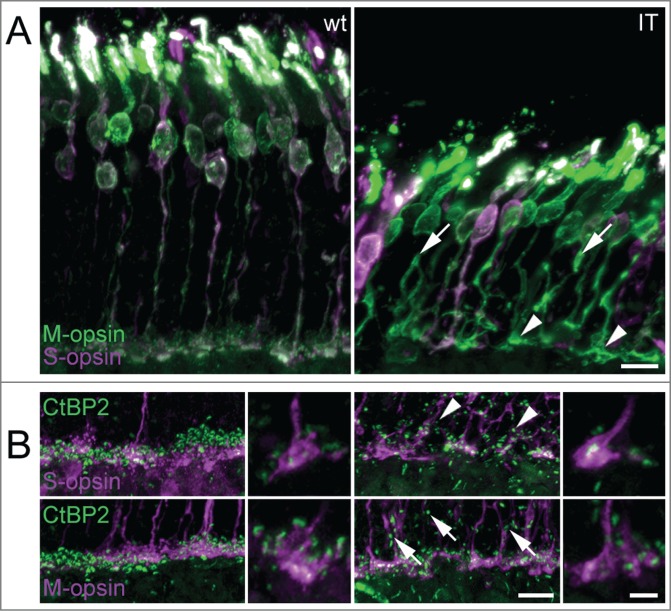
M- and S-cone morphology in wild type and Cav1.4-IT mouse retina. (**A**) Sections of transverse retina double-labeled with antibodies against M-opsin (green) and S-opsin (magenta). In Cav1.4-IT mice, both S- and M-opsin cones were shorter and structurally degenerated. Axons formed branches and showed numerous varicosities (arrows). Synaptic terminals were enlarged and misshaped (arrowheads). (**B**) Triple-staining for synaptic ribbon marker CtBP2 (green) and S- and M-cone opsin (magenta). Wild type mice showed mature horseshoe-shaped synaptic ribbons in both S- and M-cone pedicles whereas Cav1.4-IT ribbons in S- and M-cone pedicles were variable in morphology. Ectopic synapses in the outer nuclear layer (arrows and arrowheads) were more numerous in the Cav1.4-IT retina and never associated with cone markers. Scale bar 10 µm; higher magnification 5 µm.

**Figure 5.**
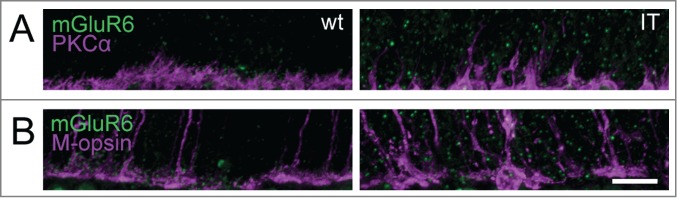
Expression of the metabotropic glutamate receptor 6 in ON bipolar cells of wild type and Cav1.4-IT retinas. Double-labeling with antibodies directed against the metabotropic glutamate receptor 6 (mGluR6, *green*) and PKCα (*magenta*) in (**A**) and mGluR6 (*green*) and M-opsin (*magenta*) in (**B**). Scale bar, 10 µm.

Our MEA experiments clearly showed altered ganglion cell responses in isolated Cav1.4-IT retinas under M-cone stimulating conditions. Because ON- and OFF-pathways in the mammalian retina separate at the photoreceptor synapse it is possible that functional alterations observed already originate at this level. The localization of Cav1.4 in cone photoreceptor terminals has been confirmed recently.^12^ In a previous study, we reported a significant decrease in cone photoreceptor length in adult Cav1.4-IT mice as determined by staining with peanut agglutinin (PNA,^10^). However, PNA labels all cones, irrespective of their spectral identity, because PNA binds to the extracellular glycoprotein matrix that is secreted by the inner segment of cones.^23^ Using S- and M-opsin specific antibodies, which label S- and M-opsin cones from their outer segments to the pedicles, we demonstrated here that all spectral types of cones present in mouse were shorter in the Cav1.4-IT retina (**Fig. 4**), consistent with the observed reduction in retinal thickness (Thickness in [µm]: outer nuclear layer: 42.9 ± 0.96 and 31.3 ± 1.34, p-value 0.0022; inner nuclear layer: 21.2 ± 0.83 and 19.4 ± 0.73, p-value 0.03095; Mann Whitney U test; for wild type and Cav1.4-IT, respectively) and confirming earlier reports.^10,13^ In ten week old mice, both S- and M-opsin cones were affected by structural degeneration. Some axons showed nodular structures and some formed collaterals (**Fig. 4A**). Most cone pedicles were enlarged, elongated and some showed an ellipsoid shape (**Figs. 4B, C** and **5A**). Immature synaptic structures, as inferred from the more elongate than punctate staining pattern obtained with the synaptic ribbon marker CtBP2, were observed in both M- and S-opsin cones (**Fig. 4B, C**). The dorsal-to-ventral gradient of M- and S-opsin expression known for the wild type mouse retina was maintained in Cav1.4-IT retinas (data not shown).

**Figure 6.**
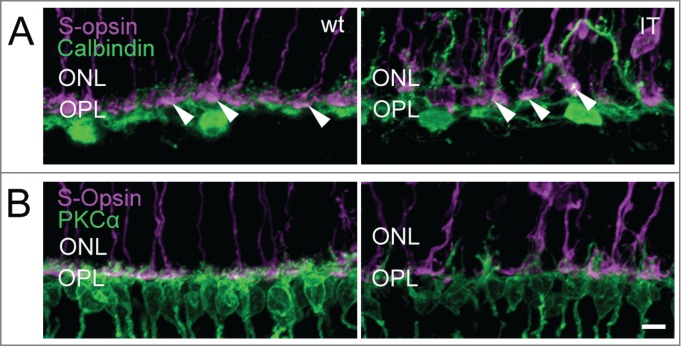
Structural integrity of horizontal cells and cone terminals of wild type and Cav1.4-IT retinas. Labeling against (**A**) S-opsin (*magenta*) and Calbindin (*green*) (**B**) S-opsin and PKCα (*green*) is shown. Calbindin staining in wild type showed puncta, corresponding to the fine dendritic tips of horizontal cells approaching the synaptic cavity of cone terminals (arrowheads). In Cav1.4-IT mice the dendrites of horizontal and rod bipolar cells were clearly elongated into the outer nuclear layer (ONL). Some connections between these 2 second order neurons and cone photoreceptor were visible (white puncta, arrowheads). OPL, outer plexiform layer. Scale bar, 10 µm.

In the Cav1.4-IT retina, PKCα immunostainings delineated the aberrant morphology of rod bipolar cells with dendrites extending deep into the outer nuclear layer (**Fig. 5A**). Staining against the metabotropic glutamate receptor mGluR6 typically expressed at dendritic tips of ON-bipolar cells (**Fig. 5A**) also pointed to a disorganization of the outer plexiform layer in Cav1.4-IT mice. The number of mGluR6 puncta at the dendrites of rod bipolar cells appeared slightly reduced in the Cav1.4-IT retina compared to the wild type. Similarly, the number of anti-mGluR6 positive puncta at the cone pedicles appeared to be reduced (**Fig. 5B**). In Cav1.4-IT retinas, the dendrites of horizontal cells extended into the outer nuclear layer.^12,13^ Double immunofluorescence labeling with CalbindinD28k and S-opsin clearly demonstrated that in the Cav1.4-IT retina horizontal cell dendrites approached both unaffected and displaced cone photoreceptor terminals (**Figs. 6A**). Rod bipolar cells did not contact ectopic cone synapses (**Fig. 6B**) as has been demonstrated in several animal models of retinal degeneration.^24,25^

**Figure 7.**
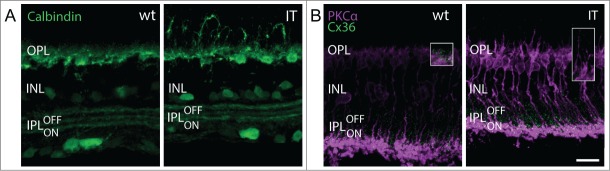
Inner retinal morphology in wild-type and Cav1.4-IT retinas. In (**A**) retinal slices were labeled with anti-calbindinD28k (Calbindin, *green*) which stains horizontal cell dendrites in the outer plexiform layer (OPL), but also amacrine cell bodies in the inner nuclear layer (INL), and amacrine and ganglion cell dendrites which are confined to 3 narrow bands in the inner plexiform layer (IPL). In both wild type and Cav1.4-IT the IPL was precisely stratified into 3 layers. (**B**) shows colabeling of PKCα (*magenta*) together with Connexin 36 (Cx36, *green*). As depicted in the insets, OPL Cx36 signal was diminished in Cav1.4 IT retinas compared to the wild type. Scale bar, 10 µm.

Immunostainings with anti-CalbindinD28k - which in mouse also labels some amacrine and ganglion cells - demonstrated that the gross layering of the inner plexiform layer was largely preserved in Cav1.4-IT retinas (**Fig. 7A**). The three characteristically distinct strata formed by certain types of amacrine and ganglion cell dendrites^26^ were present and their spacing was similar in wild type and Cav1.4-IT (**Fig. 7A**).

The gap junction protein Connexin36 (Cx36) is expressed in several neuronal classes in the outer and inner retina.^27^ Therefore we investigated Cx36 expression also in Cav1.4-IT retinas using immunofluorescence. The expression pattern of Cx36 in the inner plexiform layer was similar in wild type and Cav1.4-IT retinas (**Fig. 7B**). In contrast in Cav1.4-IT retinas, Cx36 staining was reduced in the outer plexiform layer (**Fig. 7B**, inset) where Cx36 is reported to be expressed on the cone side of the gap junction in mouse retinas.^28^ The reduction was obvious at the dendritic tips of sprouting rod bipolar cells which rarely revealed Cx36 positive dots in their close vicinity in Cav1.4-IT. Together our findings showed that the inner retina in Cav1.4-IT mice was not dramatically affected by structural alterations, and the observed functional deficits arise mainly in the outer retina.

**Figure 8.**
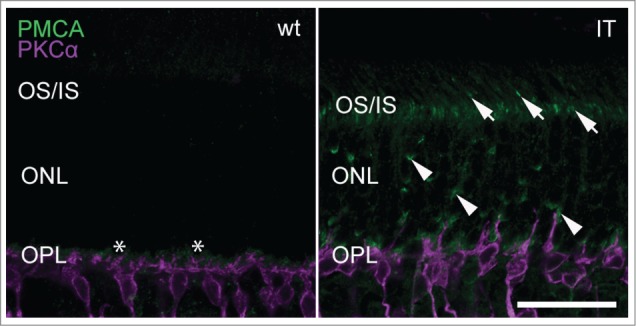
Expression of PMCA1 in wild type and Cav1.4-IT retinas. Transverse retinal slices were labeled with anti-PMCA1 (*green*) together with PKCα (*magenta*). In wild type, PMCA1 expression was typically seen in the outer plexiform layer (OPL) abundant at the site of the photoreceptor terminals, designated by the asterisk (*). In Cav1.4-IT retinas strong punctate PMCA1 staining was also observed in photoreceptor outer and inner segments (OS/IS; arrows) and in the outer nuclear layer (ONL; arrowheads). Scale bar, 10 µm.

A gain-of-function mutation affecting Cav1.4 channels in retinal photoreceptors is also expected to affect general photoreceptor metabolism. One possibility is that an excess influx of calcium challenges calcium extrusion mechanisms. In rod and cone terminals steady-state intracellular calcium is maintained by plasma-membrane calcium ATPase 1 (PMCA1) transporters. Among several isoforms present in the retina, PMCA1 is most abundant and required for calcium clearance in tonically depolarized photoreceptors and cone bipolar cells.^29^ PMCA1 expression in Cav1.4-IT retinas was prominent in the outer plexiform layer similar to wild type (**Fig. 8**).^30,31^ Interestingly, we also observed several strong PMCA1 immunoreactive puncta in the outer nuclear layer as well as the photoreceptor inner segments which were clearly missing in wild type retinas (**Fig. 8**). This suggests that PMCA1 was dislocalized in Cav1.4-IT and leaves possible that PMCA1 has a role in maintaining intracellular calcium homeostasis in photoreceptors.

## Discussion

Enhanced Cav1.4 channel activity inferred from the hyperpolarizing shift of the activation curve of heterologous expressed Cav1.4-IT channels^18^ resulted in a loss-of-control of synapse maturation.^10,12,13^ This was similar to other Cav1.4 channel mutants or mutants lacking different proteins important for formation of the first synapse in the visual pathway - the photoreceptor ribbon synapse.^11,15,17,32^ Recent reports showed that the level of synaptic maturation in adult Cav1.4-IT mice was quite variable.^10,12^ Our MEA experiments implicated that a certain amount of functional synapses is still formed in Cav1.4-IT animals. However, these only allowed anomalous downstream synaptic transmission and gave rise to a decrease in contrast sensitivity function as seen in our MEA experiments. Because most synapses in Cav1.4-IT retinas were found abnormal, elongated or roundish, these reasonably reflected the significant number of non-responding ganglion cells. The assumption was supported by the evidence that photoreceptor synapses in Cav1.4-KO mice remained mostly immature.^12,13^ Further this is in line with our observation of a non-responsiveness of Cav1.4-KO retinas to physiological light; previously predicted by the absence of visually evoked cortical activation.^8^ Interestingly, *nob2* mice were still capable to detect and follow moving sinusoidal-waves when optokinectic responses were recorded.^16^

Calcium imaging experiments demonstrated that Cav1.4-IT retinas retained some ability to modulate calcium entry in the synaptic terminals of retinal photoreceptors in response to membrane depolarization.^13^ Due to the higher channel activity at depolarized membrane potentials elevation of basal calcium levels in photoreceptors is suggested leading to increased glutamate release in darkness, and downstream, increased activity of ganglion cells upon light decrement. In our MEA recordings, a population of cells (units) indeed showed high discharge activity in absence of a light stimulus. The ‘suppressed’ ganglion cell activity observed upon light exposure pointed to an increase in the time needed to shut off glutamate release upon light exposure. This delayed onset between light exposure and ganglion cell response can also be explained by a reduction in the of photoreceptor dynamic range as suggested by the gain-of-function nature of Cav1.4.IT channels. Cav1.4 immunoreactivity was reported in bipolar cells^8,33,34,35^ therefore Cav1.4 channels may also be involved in the regulation of the release at their terminals. Our current recording approach, however, does not permit to resolve a contribution of Cav1.4 at this site. Anyhow, a simple interpretation based solely on changes in Cav1.4 channel activity will not hold true because the input that ganglion cells receive from photoreceptors is projected on them via a tightly regulated cellular network (including bipolar cells). Photoreceptors communicate with ganglion cells through horizontal (and also amacrine) cells which mediate the lateral inhibition of photoreceptor output to enhance contrast sensitivity. From horizontal cell ablation studies one can appreciate that a lack of horizontal cell inhibition would contribute to the changes in the time course of ganglion cell responses and the reduction in contrast sensitivity.^36^ We found some horizontal cells contacting cones but could not resolve whether intact triad synapses are formed with bipolar cells. Ultrastructural analyses of cone photoreceptor terminals in the same mouse model proposed that many cones contained free-floating ribbons^13^ but the possibility that in some cones structurally intact triad synapses are still preserved was not excluded. Except some pedicles that were displaced in the outer nuclear layer, the location of cone photoreceptor synapses appeared unaffected. Therefore, the abundant presence of CtBP2 positive ribbons in the outer nuclear layer strongly suggested that rod spherules are located aberrantly. Intriguingly, sprouting of rod bipolar cell dendrites has also been noted in humans with retinitis pigmentosa,^37^ in several experimental models of retinal detachment^38^ as well as in normal aging retina.^39^ Local sprouting of cones seen in the outer nuclear layer and enlarged pedicles in the outer plexiform layer (^10^, also this study) suggested that cone photoreceptors of Cav1.4-IT retinas are in the process of degeneration; similar to Cav1.4 deficient retinas^40^ and, although less prominent, human retinitis pigmentosa retinas.^37^ Likely as a consequence of photoreceptor loss we observed a reduction of mGluR6-stained sprouting ON bipolar cells in Cav1.4-IT retinas. Similarly, receptor alterations were reported as an early response to the loss of photoreceptors in retinal ablation and degeneration models.^41,42^ Furthermore Cx36 as gap junction protein^43^ seemed less expressed and could even mark a loss of cones in Cav1.4-IT retinas. It is therefore difficult to interpret whether changes in horizontal cell morphology follow cone aberrations or are a consequence of disrupted synaptic transmission during development. Because in mouse Cx36 is reported to be expressed on the cone-side of the gap junction,^28^ this finding suggests that the coupling between adjacent cones or cones and rods and therefore lateral interactions between photoreceptors may be disrupted.

Together, the gain-of-function mutation in retinal Cav1.4 channels still supported minor, though delayed retinal signal transmission, in contrast to the lack of Cav1.4 channel function. In combination with structural alterations at the synapse a reduction in contrast sensitivity was underlying the visual dysfunction seen in the animal model but possibly also in humans carrying the gain-of-function mutation. However, Cav1.4-IT mouse retinas might drive a potential compensatory system to protect against calcium overload by translocation of PMCA1 to regions important for metabolic operations. Our data further indicated that PMCA1 localization is not only compromised by the loss of Cav1.4 protein^44^ but might also depend on Cav1.4 channel activity or channel gating.

## Materials and Methods

### Animals

Animals were housed in groups of 2–6 per cage under standard laboratory conditions (12:12 light/dark, lights on at 07:00 h, 22 ± 2°C, 50 – 60 % humidity) with pelleted food and water available *ad libitum*. Experimental procedures were designed to minimize animal suffering and the number of used animals and approved by the national ethical committee on animal care and use (Austrian Federal Ministry for Science and Research).

### Cav1.4 mouse lines

We used 2 mouse models made by Dr. Marion Maw (University of Otago, Dunedin, New Zealand): i. a Cav1.4 deficient mouse (Cav1.4-KO) and ii. a model carrying the mutation I745T in the *CACNA1F* gene identified in a New Zealand CSNB2 family (Cav1.4-IT).^6^ Only male mice were investigated. Genotyping was performed as described in.^10^

### Immunocytochemistry

#### Fixation and embedding

Ten week old mice were administered isofluran (Forane®) and killed by decapitation between 8 and 10 AM. All following steps were conducted at room temperature. Eyes were quickly removed, opened at the sclera-corneal rim and immersed for 10 min in 2% paraformaldehyde (PFA) in 1x phosphate-buffered saline (1x PBS, pH 7.3). Cornea, lens, and vitreous were removed and the eye cups fixed for 10 min in 2% PFA/1x PBS, rinsed 4x in 1x PBS, and cryoprotected in 30% sucrose in 1x PBS overnight. Eye cups were infiltrated overnight in 30% sucrose in 1x PBS and OCT medium (Tissue-Tek, Sakura, 1:1), orientated along the dorsoventral axis and frozen in liquid nitrogen pre-chilled isopentane. Vertical sections (16 µm) were cut with a cryostat (Leica Microsystems, Germany), mounted on Superfrost Plus slides (Menzel Gläser, ThermoScientific) and stored at −20°C.

#### Immunolabeling

For immunolabeling indirect fluorescence was used. Sections were washed 3 times in washing buffer (1x PBS, 0.1% to 0.5% Triton X-100, 0.05% sodium azide; for mGluR6 staining sodium azide was omitted) and blocked in washing buffer containing 1 – 10% normal goat serum (NGS; Invitrogen) or 1% bovine serum albumin (Sigma-Aldrich, A7030). Sections were incubated with primary antibodies in blocking solution at concentrations listed in **Table 1** and incubated overnight at 4°C. The next day, sections were washed 3 times in washing buffer, incubated with the appropriate secondary antibody (**Table 2**) for one hour, washed 3 times and counterstained with DAPI. Aqua-Poly/Mount (Polysciences) was used for mounting.

**Table 1.** Primary Antibodies and markers investigated in immunohistochemistry

Protein or Markers / Antibodies	Host	Working dilution	Immunogen	Source, Catalog Order No.
Calbindin D-28k	Rabbit	1:10.000	Recombinant rat calbindin D-28k	Swant, CB-38a
Connexin 36	Rabbit	1:1000	C-terminal region of the human Connexin 36 protein	Invitrogen (Novex®), 36–4600
CtBP (E-12)/ RIBEYE	Mouse Monoclonal	1:500	Amino acids 1–400 of human CtBP1	Santa Cruz, sc-17759
DAPI		1:10.000	–	Sigma, D-9542
Glycogen-phosphorylase	Guinea Pig	1:200	Rat muscle-specific sequence, Amino acids 826–742	Dr. Hambrecht
mGluR6	Guinea pig	1:300	C-terminus of rat mGluR6, Sequence: AAPPQNENAEDAK	Neuromics, GP13105
M-opsin	Rabbit Polyclonal	1:200	Recombinant human red/green Opsin	Millipore, AB5405
PMCA1ATPase	Rabbit Polyclonal	1:100	Synthetic peptide corresponding to the residues A(5) N N S V A Y S G V K N S I K E A N(22) of rat PMCA1 ATPase.	ThermoScientificPA1–914
PKCα	Mouse	1:500	C-terminus of human PKCα, Amino acids 645–672	Santa Cruz,sc-8393
PKCα	Rabbit	1:500	C-terminus of human PKCα	Santa Cruz, sc-208
S-opsin	Goat polyclonal	1:200	N-terminus of human OPN1SW	Santa Cruz, sc-14363

**Table 2.** Secondary antibodies investigated in immunohistochemistry

Secondary antibodies	Working dilution	Source, Catalog Order No.
Alexa Fluor® 488 Goat Anti-Rabbit IgG (H^+^L)	1:400	Molecular Probes® A11001
Alexa Fluor® 488 Donkey Anti-Rabbit IgG (H^+^L)	1:400	Molecular Probes® A21206
Alexa Fluor 568 Goat Anti Mouse lgG (H^+^L)	1:400	Molecular Probes® A11004
Alexa Fluor 568 Donkey Anti Mouse lgG (H^+^L)	1:400	Molecular Probes® A10037
Alexa Fluor® 647 Goat Anti-Guinea pig IgG (H^+^L)	1:400	Molecular Probes® A21450
Alexa Fluor 647 Donkey Anti Goat lgG (H^+^L)	1:400	Molecular Probes® A21147

### Confocal microscopy and image analysis

Sections were imaged with a confocal microscope (Nikon, confocal A1R+) and series of micrographs were taken at 0.25 µm intervals and collapsed to a z-projection with maximum intensities in ImageJ (National Institutes of Health, Bethesda, Maryland, USA^45^). For the projection of PMCA1 labeled sections z-stacks were taken at 0.1 µm intervals. For the analysis of retinal thickness micrographs were taken with a Zeiss Axiovert 200M (Carl Zeiss). Retinal layers were measured using ImageJ, the data exported to GraphPad Prism and compared using the Mann-Whitney U test. Images were adjusted for contrast, brightness, and colors and assembled to figures using Adobe Photoshop CS5.

### Multielectrode recordings

#### Retina preparation

Adult wild type, Cav1.4-KO or Cav1.4-IT mice (age: 66 – 110 days) were dark adapted for at least 30 min. Eyes were removed and retinas were dissected in 37°C AMES medium (Sigma Aldrich, Austria) equilibrated with 5% CO_2_- 95% O_2_. Subsequently retinas were placed ganglion cell side up on a nitrocellulose filter that contained a 2 mm diameter hole and flattened. With this carrier the retina was placed ganglion cell side down on the recording field of a perforated multielectrode array (pMEA, 60 electrodes, 30 µm diameter, electrodes at 200 µm spacing; Multichannel Systems, Reutlingen, Germany) and continuously superfused with 36°C bubbled AMES medium. Slight suction through the perforated electrode field was applied using an electronically controlled vacuum pump (Multichannel Systems; Reutlingen Germany).

#### Stimulation & recording

The retina preparation was placed under an upright Microscope (Axio Examiner A1, Zeiss Germany) and the viability was controlled using a short series of light pulses. After that the retina was dark-adapted for 30 min (black screen) before starting the experiments. Visual stimuli were generated using VisionEgg^46^ and presented with a 120 Hz TFT Screen (Samsung), calibrated to a linear γ value of 1.0 using a Spyder3 colorimeter (Datacolor Imaging Solutions, USA). Stimuli were applied via the microscope back port using a photographic lens (85 mm f1.8 @f2, Nikon, Japan). The spectral range of stimuli was constrained by a 510/40 spectral filter (Chroma, USA), limiting it to a range specific for mouse rods and M-cone excitation. Via a 80/20 beam splitter (AHF Analysentechnik, Tuebingen Germany) 80% of stimulus light was passed to the preparation while 20% was passed to the oculars allowing visual control of the preparation and stimulation at the same time. Finally images were focused on the photoreceptor layer under visual inspection using a 10× NA 0.3 water immersion objective (Zeiss, Germany). One screen pixel comprised 4.5 µm × 4.5 µm on the retina. Light intensities were measured directly at the objective lens using a power meter (Thorlabs, Germany) ranging from 10 µWm^−2^ (≈25 photons µm^−2^s^−1^; black screen) to 12.7 mWm^−2^ (≈32000 photons µm^−2^s^−1^; white screen). Frame timing was monitored by a custom built photodetector directly mounted onto a corner of the screen. Ganglion cell responses were recorded at 25 kHz sampling rate using a MEA2100 amplifier (Multichannel Systems; Reutlingen Germany).

*Analysis*: Retinal spikes were extracted and sorted into single units using WaveClus^47^ with slight modifications in Matlab 2011b: signals were high-pass filtered at 200 Hz using a wavelet based approach^48^ and low-pass filtered at 3000 Hz using a zero phase-lag elliptic filter. For peri-stimulus time histogram (PSTHs) analysis the time range between 0.5 s before stimulus onset and 1.5 s after the stimulus was considered. Spike times were binned at 10 ms for each presentation. Spikes detected during 300 presentations of the same stimulus were pooled. For the analysis of response latencies PSTHs were smoothed using a Gaussian window function (SD = 30 ms). Latency of response was defined as the point where the smoothed mean firing rate crossed the threshold of 37% above background spiking rate, which was determined during the 0.5 s before stimulus onset.^19^ Contrast sensitivity was measured in response to drifting sinusoidal gratings. Gratings were presented in front of a mid gray background (6.2 mWm^−2^, ≈15000 photons µm^−2^s^−1^) after retinas were adapted to this background for 20 min. The visual angle covered by the field of view corresponded to 38.4° for an axial length of 3.3 mm^24^. Combinations of spatial frequencies (0.02, 0.06, 0.2, 0.6 and 2 c/d) with contrast values from 0.22 – 0.47 were presented 10 times each with a constant drifting frequency of 1.5 Hz for 10 s.^21^ PSTHs with a bin size of 10 ms were constructed by pooling the 10 presentations; subsequently power spectra were calculated. The stimulus was defined as detected in case the power at 1.5 Hz exceeded 5 times the mean background in the power spectrum.

### Statistics

Values are presented as mean ± SEM for the indicated number of experiments (n) from the indicated number of animals (N), unless stated otherwise. For multiple comparisons of *in-vitro* data statistical significance was determined by a one-way analysis of variance (ANOVA) followed by Bonferroni multiple-comparison or Dunnett's post-hoc test. For comparisons of 2 groups, data were analyzed by Student's t-test as indicated for individual experiments.
